# Supplementary material for: Hydrogen Gas Inhalation Improved Intestinal Microbiota in Ulcerative Colitis: A Randomised Double-Blind Placebo-Controlled Trial
Source: Biomedicines. 2025 Jul 23;13(8):1799. doi: 10.3390/biomedicines13081799 (PMC12383636; doi:10.3390/biomedicines13081799)
Supplement: Supplementary file 1 [file biomedicines-13-01799-s001.zip › biomedicines-3688797-supplementary.pdf]

Supplementary Materials

Supplementary method S1: Metagenomic Analysis

Microbial DNA was prepared using an enzymatic lysis method, as previously described [31]. Briefly, the frozen samples were thawed, suspended in 1-mL phosphate-buffered saline, and centrifuged at 12,000 × g for 10 min at 4°C. Subsequently, the pellets were washed with Tris-ethylenediaminetetraacetic acid (EDTA) (TE20) buffer (10 mM Tris–HCl, 20 mM [EDTA]) and centrifuged at 12,000 × *g* for 10 min at 4°C. Thereafter, 800 µL of TE20 was added to the pellets, followed by 15 mg of lysozyme and 2000 units of purified achromopeptidase. The samples were incubated at 37°C for 2 h with gentle mixing, followed by the addition of 1 mg proteinase K and 100 µL of 10% sodium dodecyl sulphate. Furthermore, we incubated the samples at 55°C for 1 h with gentle mixing. The DNA was extracted using phenol/chloroform/isoamyl alcohol (25:24:1), precipitated with isopropanol and 3 M sodium acetate, washed with 75% ethanol, and re-suspended in 100 µL of TE (10 mM Tris–HCl, 1 mM EDTA) buffer. Subsequently, 1 µg RNase A (final concentration 10 µg/mL) was added to each DNA sample and incubated for 30 min at 37°C with gentle mixing. The DNA was purified using a 20% polyethylene glycol solution, centrifuged to form pellets, rinsed with 75% ethanol, and dissolved in TE buffer.

The extracted DNA was fragmented into 200–300 bp using the Acoustic Solubiliser Covaris (Covaris, MA, USA). Sequence libraries were generated and adapter ligation performed using the ThruPLEX DNA-seq Kit (Clontech Laboratories, CA, USA). Approximately 150-bp paired-end reads were sequenced on the NovaSeq 6000 (Illumina) using NovaSeq 6000 S4 reagent kit v1.5 (Illumina) following the manufacturer's protocol NovaSeq 6000 sequence system guide v16 (Illumina) after purification using AMPure XP beads (BECKMAN).

Reads whose quality value (QV) were ≤ 20 and duplicated reads were removed with ParDRe and fastp (Chen, iMeta, 2023). In addition, reads with QV ≤ 20 for > half of the read length and reads whose length was <50 bp were filtered out. Human-derived and phiX reads were removed by mapping the reads to each genome (human genome [T2T] [32] and phiX bacteriophage genome) using minimap 2 (version 2.13-r850) [33]. Taxonomic profiles of the metagenomic samples were obtained with mOTUs2 version 3.0.1 [34] by subsampling 10 million reads using seqtk (version 1.2-r102-dirty). Subsequently, quality-controlled reads were assembled using MEGAHIT v1.2.9 [35] with default parameters to construct contiguous sequences (contigs).

Assembled contigs with lengths ≤500 bp were removed using seqkit (version: 2.3.0) [36]. Gene and protein sequences were predicted using the Prodigal software (v2.6.3) [37]. Both Prodigal and CD-HIT (version 4.6) [38] were employed after concatenating the genes and proteins to construct *de novo* non-redundant gene and protein sets. In addition, the *de novo* nonredundant gene and protein sequences were clustered to create

gene and protein catalogues. The created protein catalogue was annotated to the Kyoto Encyclopedia of Genes and Genomes (KEGG) database [39-41] using Kofam-scan v1.3.0 [42]. Furthermore, the gene catalogue was mapped to subsampled short reads using Bowtie 2 (version 2.4.5) [43]. Functional composition data were calculated as RPKM by combining the mapped data with the KEGG annotation results.

Association analyses between the relative abundance of taxa or KEGG orthologies and disease or clinical features were conducted using the R package 'MaAsLin2', involving only the taxa with a prevalence <0.1 [44].

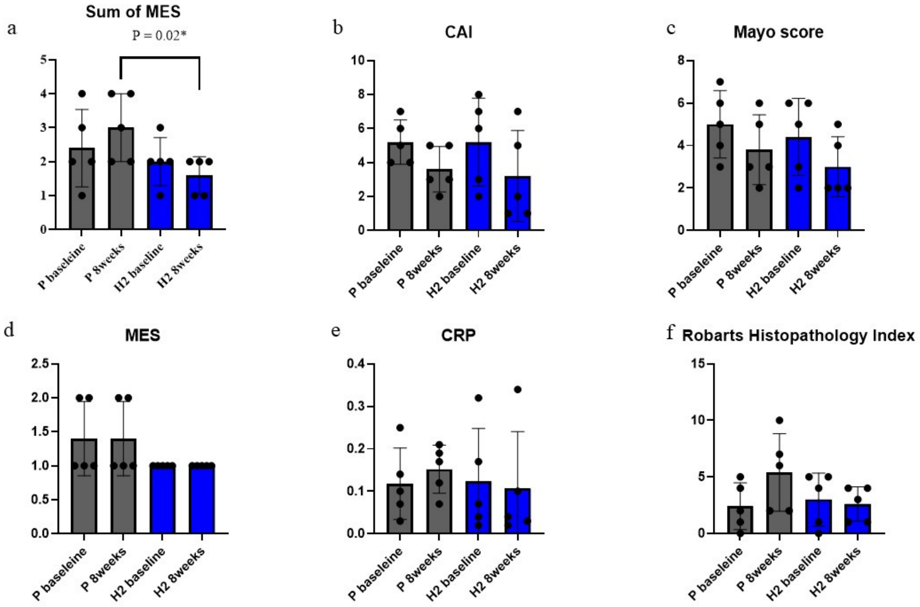

Supplementary Figure S1

Changes in sum of MES in the placebo group and hydrogen group before and after inhalation. There are no significant differences in the endoscopic scores before and after inhalation between the hydrogen and placebo groups. A comparison between the hydrogen and placebo groups after inhalation shows a significant difference in the sum of MES (Mann–Whitney U test, $p = 0.02$). There were no significant differences in CAI, MES, Mayo score, CRP level, or histological indices.

MES, Mayo Endoscopic Subscore; H_2, Hydrogen
